# An Extensive Comparison of the Clinical Efficiency of Acidulated Phosphate Fluoride (APF) and Neutral Sodium Fluoride (NaF) Oral Rinses in the Prevention of White Spot Lesions during Fixed Orthodontic Treatment: A Randomized Controlled Trial

**DOI:** 10.1155/2022/6828657

**Published:** 2022-03-20

**Authors:** Lakshmi Narayana Pilli, Gowri Sankar Singaraju, Venkatesh Nettam, Thejasree Keerthipati, Prasad Mandava, Anand Marya

**Affiliations:** ^1^Department of Orthodontics, Narayana Dental College, Nellore, Andhra Pradesh 524003, India; ^2^Department of Orthodontics, Faculty of Dentistry, University of Puthisastra, Phnom Penh 12211, Cambodia

## Abstract

**Background:**

The purpose of this randomized trial is to compare the efficacy of weekly once regime of neutral sodium fluoride (NaF) oral rinse with that of acidulated phosphate (APF) formulated daily mouth rinse in the reduction of white spot lesions (WSLs) associated with fixed orthodontic appliance treatment.

**Methods:**

The participants (*n* = 90) of this single-center, two-arm parallel study without a control group were randomly assigned with 1 : 1 distribution to each of the two groups after the bonding of brackets. Group A/test group 1 (*n* = 45) was given weekly rinse of neutral sodium fluoride (*Colgate® PreviDent® Dental Rinse-*0.2% NaF), and for group B/test group 2 (*n* = 45), an APF formulated daily oral rinse (*Colgate® Ortho Defense^@^ PhosFlur® Rinse*-0.044% w/v of NaF) was given for six months. The outcome was assessed by the International Caries Detection and Assessment System (ICDAS) index for scoring the demineralization, and for scoring gingivitis, Loe and Silness gingival index (GI) was utilized. Four different time points “T0”immediately before bonding procedures, “T1” after 4weeks, “T2” after 12 weeks, and “T3” after 24 weeks were taken to assess the ICDAS and GI scores.

**Results:**

The mean ICDAS scores for group A (NaF) were 0.025, 0.051, 0.093, and 0.113 and for group B (APF) were 0.014, 0.022, 0.038, and 0.015 at different points of time. The GI scores for group A were 0.008, 0.22, 0.33, and 0.38 and for group B were 0.003, 0.136, 0.181, and 0.097 at different time points. There was a statistically significant difference (*p* < 0.05) for both groups in terms of reducing WSL and GI.

**Conclusion:**

APF formulated daily oral rinse—0.044% w/v of NaF—is more effective than the weekly once regimen of 0.2% NaF oral rinse to prevent white spot lesions.

## 1. Introduction

As a sign of initial enamel demineralization and subsequent caries, white spot lesions are common undesired side effects during orthodontic treatment with fixed appliances if oral hygiene is not maintained. A white spot lesion (WSL) is defined as a “subsurface enamel porosity from carious demineralization,” and its formation is the initial stage of the caries process [1]. White spot lesions compromise the health of dentition and orthodontic esthetic results. The demineralization of enamel gives a whitish appearance to the WSL due to the loss of translucency of the enamel. The tooth surface may feel rougher than usual when checked with a sharp instrument through cavitation is not seen [2, 3]. Reasons for the higher incidence of WSL in orthodontic patients are the treatment associated restrictions, ineffective dental hygiene and increased retention of pathogenic biofilm (dental plaque) due to the presence of brackets and synthetic bonding materials, and the protective cementing medium that has been washed out around the bands [3–5]. WSL has been reported to occur on all teeth, mainly on the maxillary laterals and mandibular canines, along with premolars [6]. Among the patients undergoing orthodontic treatment, the prevalence of WSL was noted to be 2-96%, depending on the detection method. Within four weeks, visible white spot lesions were detected without any fluoride supplementation and were more prevalent in the first six months of the start of orthodontic treatment [6–8]. The WSL was noted irrespective of whether self-ligating or conventional brackets were used [9].

As described by Silverstone [10], this subsurface lesion has four distinct zones of a carious lesion in enamel: the body of the lesion, the entire surface (subsurface) area, the dark zone, and the translucent zone leading edge of the lesion. The subsurface lesion develops through a cyclic demineralization/remineralization process. Pathogenic biofilm may cause enamel demineralization and inflammation of the gingiva [11]. The various prophylactic regimes include antimicrobial agents or agents that disrupt the plaque formation or aid in remineralization of the lesions. The nonfluoride methods include chlorhexidine mouthwash [12], casein phosphopeptide-amorphous calcium phosphate (CPP-ACP) [13], resin sealers [14], and adhesives [15]. On the other side of the spectrum of prophylactic agents are the fluoride supplements which have an important role in preventing the formation of WSL topical fluorides in the form of fluoride gels [2], mouth rinses [2, 12], and varnish with different ppm of fluoride [14–17], at any dose, frequency, duration, or method of administration have been investigated. Earlier studies have shown that regular use of fluoride in toothpaste alone does not inhibit the development of WSLs around the brackets [18]. Using solvents (mouth rinse) with neutral sodium fluoride (0.05% or 0.2%) during orthodontic treatment along with the fluoridated dentifrice could reduce the demineralization process and have proven to be effective in preventing gingivitis also [18, 19].

Recently, acidulated phosphate fluoride (APF) formulated oral rinse with a low concentration of 0.044% w/v of NaF (*Colgate® PhosFlur® Rinse*) has been introduced into the market specifically for prevention and reduction of white spot lesions in orthodontic patients with fixed appliances. The suggested mode of rinse is once daily (manufacturer's instruction) for the first six months. No studies were available to verify the efficacy of this oral mouth rinse in preventing white spot lesions in orthodontic patients undergoing fixed appliance treatment. So this study is mainly aimed at evaluating and comparing the efficacy of 0.044% w/v of NaF, acidulated phosphate fluoride (APF) formulated oral rinse (*Colgate® Ortho Defense® PhosFlur® Rinse*) with that of weekly oral rinse, and neutral sodium fluoride (0.2% NaF) (*Colgate® PreviDent® Dental Rinse*) in the reduction of WSL in the early stages of orthodontic treatment. The secondary objective was to compare in terms of the reduction of gingivitis in orthodontic patients. A null hypothesis was put forward that there is no difference in the efficacy of acidulated phosphated fluoride rinse and neutral sodium fluoride rinse in preventing and reducing the initial enamel lesions and improvement in the gingival status in orthodontic patients undergoing fixed appliance therapy.

## 2. Materials and Methods

This prospective clinical study was carried out in the Department of Orthodontics and Dentofacial Orthopedics, Narayana Dental College, Nellore, Andhra Pradesh, India, from 1st September 2018 to 31st August 2019. The protocol for this clinical study was reviewed and approved by the institutional ethical committee at Narayana Dental College, Nellore. Andhra Pradesh, India (Regd. No. D178408008; Ref No. NDC/IECC/ORT/12-17/02 dated 06/12/2017).

### 2.1. Sample Size Determination

A minimum of 90 participants was required as a sample for this study. This study had two treatment groups as parallel arms with APF as test and NaF without any control groups. Accordingly, a sample size of 45 participants per treatment group will provide ≥80% power with a 5% error and 95% confidence interval (CI). This sample size is sufficient to detect a minimum difference of “1” between any two scores when measured with International Caries Detection and Assessment System (ICDAS) index [20] and gingival index [21].

### 2.2. Study Population Enrollment

The participants were recruited from a cohort group of consecutive patients registered for fixed appliance orthodontic treatment. The participants were given verbal and written explanations in English and their known naive language about the study. Informed and written consent was obtained from all the participants and potential consent from the parents/guardians if the participant is a minor.

### 2.3. Safety of the Participants

**Figure 1:**
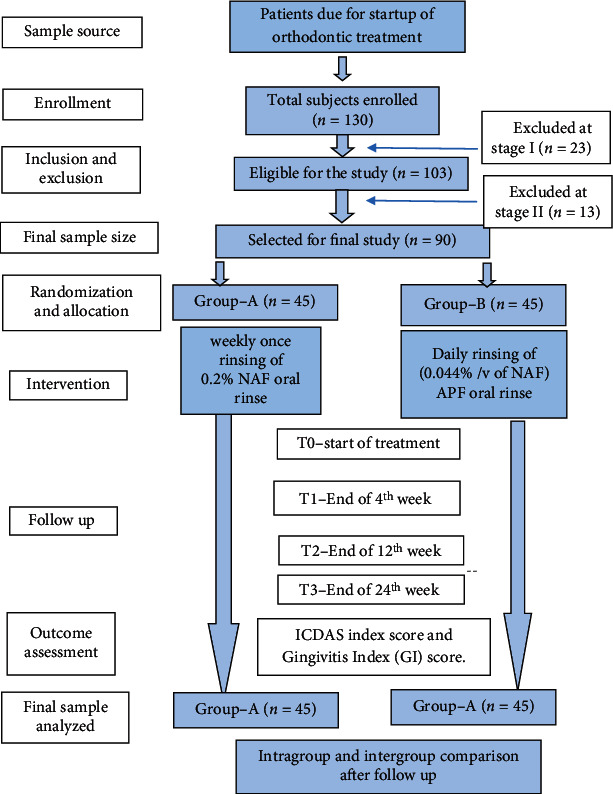
Flow chart of the study design.

The materials, methods, interventions, and protocols that were previously applied and verified as safety standards were followed in this study. The design of the study was shown in the CONSORT flowchart (Figure 1).

### 2.4. Screening Procedure

The participants were included in the final study in a two-stage step of screening the patients. A “consecutive consenting sample” was drawn from all the patients aged 12-18 years scheduled to receive fixed orthodontic treatment (buccal technique). They were informed about the study, and all those who consent and willing to participate in the trial were evaluated for eligibility. In the first step, one hundred and two (102) patients who met the inclusion and exclusion criteria were selected out of the one hundred and thirty (130) consecutive patients enrolled for the trial.

First step

Inclusion criteria are as follows:

1. All adolescent patients aged 12-18 years scheduled to receive fixed orthodontic treatment (buccal technique)
2. Patients without any detectable clinical caries risk

Exclusion criteria are as follows:

1. Dental surface with an International Caries Detection and Assessment System (ICDAS) code ≥ 2 at baseline
2. Presence of filling and restoration, oral, systemic, metabolic, or mental disease
3. Existing medication, alcohol abuse, nicotine, or drug consumption
4. Periodontitis or periodontal disease; syndrome; and cleft lip, jaw, and palate

In the second step of selection, patients with good compliance and motivation after proper instructions and reevaluation as defined by, who adhere to weekly prophylactic visits before treatment, were considered for the study. Four weeks before the start of orthodontic treatment, all the initially selected participants received professional prophylaxis with fluoride-free polishing paste and were given detailed one-on-one oral hygiene instructions. This protocol consists of manual tooth brushing with fluoride toothpaste of 1100 ppm F^−^ (*Colgate max fresh™*, *cool mint* NaF 0.24% (0.15% w/v *fluoride* ion)) for 2 min twice a day and cleaning of the interdental spaces with dental floss or an interdental brush. Participants were recalled weekly to assess gingival status for the next three weeks.

Second step

Inclusion criteria are as follows:

1. All the patients with a regular weekly periodic check-up for all the three successive appointments
2. ICDAS < 1

Exclusion criteria are as follows:

1. Were low compliance and motivation to conduct sufficient dental hygiene after proper instruction and reevaluation as defined by the failure to adhere to weekly prophylactic office visits before treatment (missing more than one)
2. Weekly prophylactic office visits before treatment (missing more than one)
3. Silness/Loe gingival index [21].of ≥1.0 at the start of orthodontic treatment (baseline)

In the second selection step, patients with good compliance and who regularly adhered to the three successive weekly prophylactic visits were finally considered for the study. Thus, the final sample included 90 participants eligible for the study. The participants selected for the trail (*n* = 90) were randomly and equally assigned to either of the two groups (*n* = 45) by the Fish-bowl method [22]. After a brief formal training about the procedure, the nursing staff (G.K.) carried out the randomization and allocation. The allocator was not aware of the purpose of the study. The individual mouth rinses were dispensed in a plain plastic bottle and calibrated cup without any label indicating the contents. After the intervention, the outcome was measured by an assessor (SGS) blind to the allocation. Though basically a single-blinded study, it was made as a double-blind procedure as the participant, and the score assessor was blinded to the group allocation. The primary investigator (PLN) could not be blinded to the interventions applied as he was the person directly involved in coding and decoding the participants and interventions of the individual groups.

### 2.5. Groups and Interventions Applied

*Group A/test group 1 (n* = 45): weekly once rinse of neutral sodium fluoride (0.2% NaF) oral rinses (*Colgate® PreviDent® Dental Rinse*).

*Group B/test group 2 (n* = 45): daily mouth rinse with acidulated phosphate (APF) formulated oral rinses 0.044% w/v of NaF (*Colgate® Ortho Defense^@^ PhosFlur® Rinse*).

*Outcome exposure measured*: primary outcome is the measurement of enamel demineralization, and the secondary outcome assessment is the measurement of the gingival health status.

*Method of assessment of outcome*: primary outcome was assessed by the ICDAS index, and the secondary outcome was assessed by the Loe and Silness gingival index (GI).

*Period, frequency, and interval period of the outcome assessment*: the timing of the evaluation was as follows: T0: day 1—before bonding-preintervention, T1: day 28—at the end of 4th week, T1: day 84—at the end of 12th week, and T1: day 168—at the end of 24th week. At all the time points, ICDAS score and gingival index were assessed.

*Assessment of white spot lesions*: the dentition from the second premolar to the second premolar in both the upper and lower arches was assessed for the demineralization status by using the ICDAS scoring criteria by visual examination as mentioned in Tables 1 and 2.

### 2.6. Examiner Calibration

Initial training was provided to three investigators (SGS, MP, and PLN) to assess the ICDAS scores by an experienced professor who had formal training in ICDAS assessment and was involved in similar research activities. Calibration of the three examiners was performed from May 1 to May 20^th^, 2018. The individual examiners' assessment of the ICDAS scores was done on ten patients under the instructor's supervision. These patients were not the participants of the present study. Initially, all the examiners assessed five patients at three different points in time. The accuracies and errors of the three examiners were compared and discussed. Another five patients were examined after one week by all three examiners. The same group of patients was reexamined after three days. The scores obtained by the examiners were compared to calculate Kendall's coefficient of concordance (Kendall's *W*) for interexaminer and intraexaminer reproducibility. The Kendall's *W* values above 0.90 were observed for all the examiners. Thus, it validated the concordance of intra- and interexaminer scores. However, in the final trial, all the measurements were performed by a single examiner (SGS). The gingival index was assessed under the supervision of an experienced periodontics professor who was blind to the study.

### 2.7. Clinical Procedures

The status of white spot lesions (WSLs) and gingival health status of the final sample of participants (*n* = 90) were initially assessed on the day of the start of orthodontic treatment—day 1 (T0). The WSL scoring was done with ICDAS scoring and gingival health by gingival index (T0). This was followed by bonding procedure where the etching was done using 37% phosphoric acid for about 15-20 seconds (*restorite etching gel containing silica and 37% phosphoric acid, prime dental products Pvt Ltd, Thane, India*), application of bonding agent and primer (*Orthosolo, universal bond enhancer, Ormco corporation, India*), and curing for about 20 seconds (*Mini S curing light Guilin woodpecker, instruments Pvt Ltd*) followed by 0.22 MBT bracket placement (Unitek TM Gemini metal brackets, 3M Unitek orthodontic products, USA) with composite (*Enlight*light cure adhesive, Ormco corporation, Italy). After placing the bracket in a correct position, excess composite around the bracket was removed using a bracket placer sickle and then light-cured for 45 sec. Then, the first archwire was placed as per the case. This was followed by randomization and equal allocation to either of the groups (*n* = 45). The intervention in group A included the 0.2% NaF agent as mouth rinse weekly once regimen, and group B received 0.44 w/v% of APF-based daily mouth rinse as per manufacturer's recommended use. After brushing the teeth, the patients were instructed to vigorously swish their mouth with two teaspoonfuls (10 ml) of the solution for 1 min and spit it out. They were directed not to swallow the mouth rinse. The patients were further instructed not to eat or drink for 30 min after rinsing. All the patients were asked to follow this regimen until their next successive appointment, after the 28th day (T1). The participants were reminded of their regimen by an automated message system. Follow-up and recall visits: as a part of orthodontic treatment procedures, these patients were recalled regularly for follow-up every month, but the outcome assessment was done at the end of 4th week (T1), 12th week (T2), and at the end of the 24th week (T3). The status of the WSL and gingivitis was recorded using the same procedure as was done at the “T0” stage before any orthodontic maneuvers were taken up at the respective stage.

*Assessment of ICDAS*: the archwire was removed at each appointment. The teeth were cleaned before examination using prophylaxis paste and brush. This was followed by visual examination of the teeth under standard light conditions on the dental chair. The lesions were coded according to the “ICDAS” criteria. The facial surfaces of maxillary and mandibular teeth from the right second premolar to the left second premolar were examined. The mesial, gingival, distal, and occlusal surfaces of the tooth adjacent to the bracket surface were examined initially under wet conditions. Teeth were isolated with cotton rolls, air-dried for 10 seconds, and then reexamined. The highest severity code of each tooth is taken as the individual score for that tooth. The scores for all the teeth were summed up to reflect the average score of each of the individual participants.

*Assessment of gingival index*: for scoring the gingival index, the tooth was divided into four surfaces, mesiobuccal, buccal, distobuccal, and the palatal surface. For calculating the gingival index, the scores for each tooth were summed up and divided by the number of surfaces examined. The final gingival score of the individual was calculated by taking the average of the scores obtained per tooth, and that of all the teeth were examined from the second premolar to second premolar in both the arches.

### 2.8. Statistical Analysis

All the data assembled was entered into Microsoft excel sheet and then statistically analyzed with a Statistical Package for the Social Sciences (*IBM SPSS version* 21 for Windows, IBM Corp released 2012 Armonk, NY). The ICDAS and GI were calculated on ordinal data but converted into continuous data by taking the average for each individual. The data is regular, quantitative, and continuous. Basic descriptions were presented in the form of mean and standard deviation, and a parametric test—Student's independent *t*-test—is used as an inferential statistical test for analyzing the difference between NaF and APF mouth rinse groups at different time intervals. Repeated measured ANOVA was used to analyze the intragroup differences at different time intervals. Intrapair differences between the different periods within the groups were analyzed by post hoc LSD-Bonferroni test. The probability (*p*) value for statistical significance was 0.05 or less for the difference between any two groups for all the analytical tests.

## 3. Results

**Figure 2:**
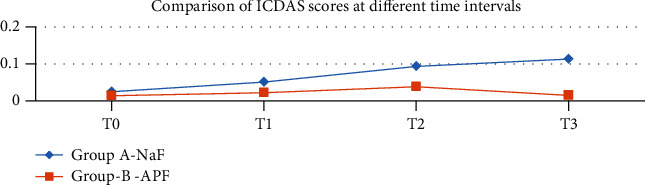
Comparison of ICDAS scores at different time intervals.

**Figure 3:**
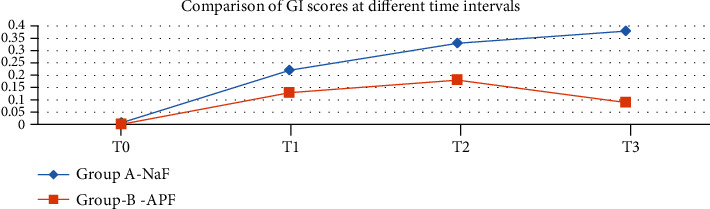
Comparison of GI scores at different time intervals.

**Figure 4:**
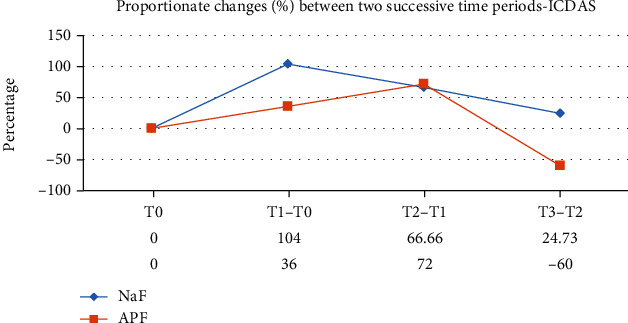
Comparison of proportionate changes (%) between two successive time periods—ICDAS.

**Figure 5:**
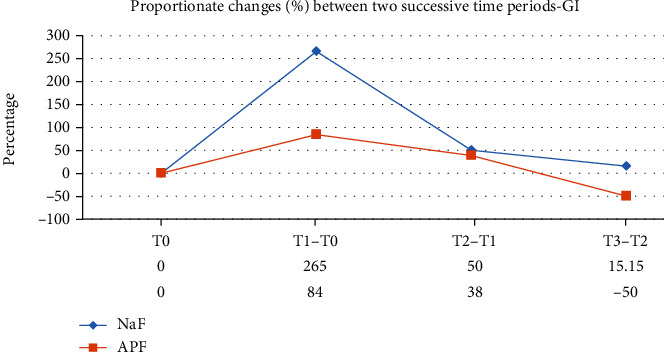
Comparison of proportionate changes (%) between two successive time periods—GI.

A total of 90 participants were evaluated (male 44 and female 46) in the final run. The mean age of the NaF group and APF group was 14.7 ± 1.08 and 15.1 ± 1.16, respectively. There was uniformity of distribution between the two groups by age and gender-wise (Table 3). The descriptive data for both the groups—mean ICDAS index and gingival index (GI)—at different time points is shown in Table 4. There was a gradual increase in the mean values from T0 to T3 through T1 and T2 in the NaF group (Table 4 and Figure 2). In the case of APF, the ICDAS and GI scores increased from T0 to T2 but declined at T3 (Table 4 and Figure 3). A repeated measured ANOVA determined that mean ICDAS and GI scores differed statistically significantly between time points in both the groups (Tables 5–8). Pair-wise comparison by post hoc tests using the Bonferroni correction revealed that in the NaF group, there was a statistically significant change in the ICDAS and GI scores between successive periods (*p* < 0.001) except for that between T2 and T3 (Tables 5 and 7). The difference in values for group B APF solution is not statistically significant between T1 and T2 for ICDAS (*p* = 0.178) and GI scores (0.245) (Tables 6 and 8). The difference in the ICDAS and GI scores between T2 and T3 is statistically significant (*p* < 0.001) (Tables 6 and 8). The ICDAS and GI scores were compared between the groups (Tables 9 and 10). Except at the baseline (T0), the difference in The ICDAS and GI scores between the groups is statistically significant at all time points (Figures 4 and 5).

## 4. Discussion

WSL is a subsurface lesion that develops due to imbalances in the dynamic homeostasis mechanism of the demineralization/remineralization process. The enamel is maintained in dynamic equilibrium with the surrounding environment, the saliva. Under normal conditions, the enamel atmosphere is supersaturated with hydroxyapatite, and a chemical equilibrium exists. A drop in pH of saliva below the critical pH of 5.5 results in demineralization as the calcium and phosphate ions diffuse out of the enamel into the surrounding environment. At acidic pH, the supersaturated saliva becomes undersaturated, and the solubility of the enamel increases. The minerals from the subsurface then replenish the mineral content of the surface enamel [22]. However, if long periods of demineralization overlap the shorter periods of remineralization, cavitation of the enamel surface ensues. The primary source of the acidic environment is initiated by organic acids released by acidogenic bacteria, such as *S. mutans* and *Lactobacilli*, located in the dental plaque. There is an overall increase in the oral bacterial count after orthodontic appliances and cariogenic bacteria [24, 25]. Previous studies have reported increased adhesion of bacteria to orthodontic devices, especially in the presence of excess resin at the bracket/tooth interface [26]. Orthodontic appliances make oral hygiene practices more complex, leading to accelerated plaque accumulation on the tooth surfaces and predisposing the enamel to demineralization and formation of WSL [27].

Patient education, extended oral prophylaxis program, sealant application, and fluoride administration have all been introduced as methods to prevent WSL formation [28, 29]. The problem with sealants is that they undergo mechanical wear, erode over time, and have to be reapplied. The most effective means of caries prevention is fluorination of the tooth through topical fluoride in the form of a toothpaste or varnish fluoride therapy [27]. When enamel is exposed to ionic fluoride present in the supplements, it may be taken up to form fluorohydroxyapatite (FHA) when the fluoride concentration is low (<50 ppm), and an acidic environment is present. The calcium in HA crystals is displaced by fluorine, forming FHA, which has a much lower solubility than the HA. The presence of fluoride in an acidic environment reduces the dissolution of calcium hydroxyapatite, and hence, there is inhibition of the demineralization of enamel. FHA has two main advantages over HA; first, fluoride acts as a catalyst, assisting in remineralizing enamel with phosphate ions dissolved in saliva [30]. This can help to counteract any demineralization which has occurred. Second, the displacement of hydroxide with fluoride removes a weakness in HA to lactic acid; FHA (Ca10 (PO4)6 F2) is relatively resistant to dissolution in an acidic environment [31].

Prolonging the exposure time or using a fluoride solution with low pH can increase calcium fluoride (CaF2) formation. Calcium fluoride (CaF2) is formed when the fluoride concentration is maintained at concentrations greater than 100 ppm. [32–34]. CaF2 formed at low pH contains less internal phosphate, which is less soluble. The CaF2 may serve as a pH-controlled fluoride reservoir, available for remineralization or inhibiting demineralization during a carious attack. Therefore, acidulated fluoride formulations provide more calcium fluoride to the enamel within a short period than neutral NaF. When APF fluoride is applied, calcium fluoride builds up in plaque, on the tooth surface, or in incipient lesions. The cariostatic effect results from the absorption of phosphate ions and protein molecules onto the calcium fluoride.

Acidulated phosphate fluoride (APF) was introduced in 2008 by Rios et al. [32]. Their study concluded that the fluoride component from acidic phosphate solutions is readily available for uptake; the results were confirmed by Saxegaard and Rolla [35]. APF solutions contain 1.23% (12,300 ppm) fluoride ion. At a low pH of 3.0, more than half of the fluoride will be in the form of hydrogen fluoride rather than free form. The studies by Wiegand et al. [36] determined that acidulated fluoride gel's ability to protect demineralized enamel against subsequent demineralization increased with increasing concentration (up to 1.25%) of the applied gels. Recently acidulated phosphate fluoride (APF) formulated oral rinse—0.044% w/v of NaF (*Colgate® PhosFlur® Rinse*)—has been introduced into the market. Luccese and Gherlone [37], for instance, could show that the first 6 months are of particular importance in the development of WSL because the adolescent patients have to adapt their hygienic practices to the requirements of orthodontic treatment. In this context, it was of interest that the present double-blinded randomized control trial was carried to evaluate the efficacy of APF mouth rinse in comparison with the weekly once 0.02 NaF mouth rinse regimen during the first six months after the startup of fixed orthodontic treatment.

In the present study, there was a mean increase of ICDAS and GI scores from T0 toT2 in both the groups (Table 4). It was observed that within the first month of orthodontic treatment, the incidence of enamel demineralization (WSL) and initial caries was significantly increased in both groups. The results also showed that WSL could not be prevented in total in all patients and were concordant with the previous study that demonstrated that even in the case of appropriate dental hygiene with fluoride toothpaste and the use of additional preventive measures, such as the application of fluoride varnish, the WSL could not be prevented in toto [36]. The most probable reason was the presence of plaque predilection sites caused by the fixed appliance that impeded effective cleaning and plaque removal utilizing domestic dental care.

In the present study, an increase of 104% in ICDAS scores was observed at the end of 1 month (T1) in the NaF group, and at the same time, there was only a 36% increase in the APF group compared to baseline scores (T0) (Figure 4). However, in the present study, there was a drop -60% between T2 and T3 periods of the APF group, which is statistically significant (*p* < 0.001) (Tables 4 and 5 and Figure 2). Though there was a proportionate decrease between T2 and T3 compared to T2 and T1 in group A, an ascending slope of the ICDAS scores was still observed (Tables 4 and 6 and Figure 3). The same trend for the GI scores was observed during the given time intervals (Tables 4, 6, and 8). A sudden spike in the proportionate GI scores was observed for both the groups (265% and 84%) in the first one-month period (Figure 5).

Further, the proportionate increase in ICDAS scores and GI index remained low in APF compared to the NaF during most of the time intervals under study, and the difference was statistically significant (Tables 9 and 10). The results were in partial agreement with the study of O'reilly [2], who demonstrated that measurable demineralization occurred around orthodontic appliances after only one month. These observations of the current trial indicate that it is essential to evaluate and follow up on the patient's oral hygiene status considering the higher severity scores of WSL in the initial first month of the treatment itself. This increase may be due to an increase in the lesion's frequency or may be due to an increase in the severity of the lesion. If necessary, the preventive measures should be immediately started along with the treatment procedures. However, in the time interval between T3-T2, there is a downward trend in group B APF mouth rinse scores between the 12^th^ and 24^th^ weeks. This may indicate the phase of remineralization that was set earlier in the case of participants exposed to APF mouth rinse.

In orthodontic patients and patients with a high risk of caries, it has been suggested that fluoride dentifrices should be supplemented with a fluoride mouth rinse solution [18, 19, 38, 39]. The most commonly used concentration for adults and the elderly is 0.2% sodium fluoride (NaF) (900 ppm F) for weekly rinse or in some countries for daily use in adults and in children. However, lower concentrations (0.05% NaF, 225 ppm F) were recommended [40]. The initial interaction of relatively high fluoride concentrations with the enamel surface and plaque during application and the maintenance of proper fluoride concentration in oral fluids after the application is essential for the effective action of the topical fluorides. Recently, APF-based mouth rinses were available to be supplemented with fluoride toothpaste for control of WSL in orthodontic patients, and their efficacy has not been tested in earlier studies. Unfortunately, no earlier studies with similar interventions were available to compare the results of our trial obtained with APF mouth wash in orthodontic patients [41]. The present study implies that fluorides based on APF formulations for daily oral rinses were more effective in preventing WSL and plaque-induced gingivitis than the neutral sodium fluoride weekly oral rinses. Thus the null hypothesis stands rejected.

International Caries Detection and Assessment System (ICDAS) [20] used in the study integrates several new criteria systems into one standard system for caries detection and assessment. The criteria were also found to have discriminatory validity in analyses of social, behavioral, and dietary factors associated with dental caries. ICDAS platform was found to be practical and valid. The present study was designed with adequate power to demonstrate a statistical difference between interventions, double-blind, randomized with allocation concealment and masking of the outcome assessment. None of the patients reported any untoward effects of fluoride delivery methods used in the study. There is concern about the fluoride mouth rinse application on archwires' mechanical and surface properties based on nickel and titanium alloys. However, most of the evidence on titanium-based alloy wires was derived from *in vitro* research simulating the oral environment. The effects shown have not been validated in vivo since the only available evidence on intraorally fractured nickel-titanium archwires did not support the implication of hydrogen embrittlement as a failure mechanism [42].

### 4.1. Limitations of the Study

However, there were certain limitations inherent in the study due to the interventions and groups selected. This was a two-arm parallel study with both arms representing active interventions. There is a lack of additional control groups with no intervention to compare the results of test groups because of ethical concerns involved during orthodontic treatment regarding patient care. The present study included only the patients with a low to moderate caries risk. Thus, we could not evaluate the possible protective effects of the fluoride rinses in patients with a high caries risk or insufficient dental hygiene. The baseline data shows the uniform distribution of age and gender between the groups, but the other confounders, such as diet, brushing methods, dexterity of the participants, and fluoridated water supply, were not included in the trial design. The fluoride delivery methods in the study were dependent on patients' compliance. Noncompliant patients were excluded in the first stage of sample selection, and hence, there were no dropouts in this trial. Only two patients reported bracket failure on one of the upper premolars at the end of T1 in one of the groups. Further, the study relied on the subjective assessment of WSL status rather than the objective methods. The fluoride rinses used in the study were formulated to be used at different frequencies of self-application. Also, it must be reported that previous studies have demonstrated that in vitro application of fluoride can lead to the deterioration of the surface properties of NiTi wires. Further studies are needed to look into this aspect of fluoride application and care must be taken to recommend these products to patients who have NiTi wires placed at that point in time [43]. Further studies are suggested to compare the efficacy of APF mouth rinses and NaF mouth with the same frequency of application. Also, newer methods such as Fluorescence Induced Theragnosis can be used to compare findings between various fluoride-releasing agents [44]. In terms of remineralizing the enamel, one effective method that has been demonstrated previously is the use of 45S5 bioglass which was shown to form brushite crystals on treated enamel surfaces [45, 46]. As can be seen from the limitations, there is a definite potential to explore the role of various fluoride-releasing agents against white spot lesions and demineralization. Also, a recommendation would be to further study bioactive agents and their benefits in remineralizing the enamel affected by fixed orthodontic attachments. As orthodontists, our aim should always be to offer patients undergoing treatment better results and a good quality of life with minimal adverse effects [47]. This requires constant exploration of better techniques, materials, and methods to enable us to offer our patients the highest quality of treatment [48, 49].

## 5. Conclusion

A sixth-month observation period to compare the efficacy of acidulated phosphate fluoride (APF) formulated oral rinses and neutral sodium fluoride (0.2% NaF) for preventing white spot lesions and gingivitis revealed that APF oral rinse is more effective in the prevention of white spot lesions and gingivitis during early stages of orthodontic treatment by fixed appliances and can be included in primary preventive care program.

## Figures and Tables

**Table 1:** Classification of the carious status based upon the International Caries Detection and Assessment System (ICDAS) [23].

Score	Criteria
Code 0	Sound tooth surface
Code 1	Pits and fissures or smooth tooth surfaces
Code 2	The distinct visual change in enamel
Code 3	Localized enamel breakdown because of caries with no visible dentin or underlying shadow
Code 4	Underlying dark shadow from dentin with or without localized enamel breakdown
Code 5	Distinct cavity with visible dentin
Code 6	Extensive distinct cavity with visible dentin

**Table:** (a) Scoring criteria for gingival index (GI)

Score	Criteria
0	No inflammation
1	Mild inflammation with a slight change in the color and slight edema with no bleeding on probing
2	Moderate inflammation and edema with bleeding on probing
3	Severe inflammation and marked edema, ulceration with a tendency for spontaneous bleeding

**(b):** 

Score	Condition
0.1-1	Mild gingivitis
1.1-2	Moderate gingivitis
2.1-3	Severe gingivitis

**Table 3:** Age and gender distribution of the groups.

	Group A		Group B		*p* value
	(NaF group)		(APF group)		
*n*	45		45		
Age^∗^ (in years)	14.7 ± 1.08		15.1 ± 1.16		0.062
Gender^∗∗^	Male	Female	Male	Female	0.833
	21	24	23	22	

^∗^Unpaired Student's *t*-test; ^∗∗^chi-square test.

**Table:** (a) ICDAS scores

Time point	Group A: NaF (*n* = 45)					Group B: APF (*n* = 45)				
	Min	Max	Mean	S.D.	Percentage increase wrt previous time point	Min	Max	Mean	S.D.	Percentage increase wrt previous time point
T0	0	0.08	0.025	0.02	0	0	0.08	0.014	0.02	0
T1	0	0.21	0.051	0.03	104	0	0.08	0.022	0.02	36
T2	0.03	0.21	0.093	0.04	66.66	0	0.15	0.038	0.04	72
T3	0.04	0.36	0.113	0.06	24.73	0	0.14	0.015	0.02	-60

**Table:** (b) GI scores

Time point	Group A: NaF (*n* = 45)					Group B: APF (*n* = 45)				
	Min	Max	Mean	S.D.	Percentage increase wrt previous time point	Min	Max	Mean	S.D.	Percentage increase wrt previous time point
T0	0	0.083	0.08	0.01	0	0	0.041	0.03	0.01	0
T1	0	0.833	0.22	0.18	265	0	0.416	0.13	0.16	84
T2	0.083	0.833	0.33	0.19	50	0.042	0.375	0.18	0.09	38
T3	0.083	3.75	0.38	0.25	15.15	0	0.5	0.09	0.11	-50

ICDAS (International Caries Detection and Assessment System) index; S.D.: standard deviation; Min: minimum; Max: maximum; T0: 0; T1: 4 weeks; T2: 12 weeks; T3: 24 weeks.

**(a):** 

	Mean	Std. deviation	ANOVA *F* value	*p* value
T0	0.025	0.02665	38.553	0.001
T1	0.051	0.03765		
T2	0.0932	0.04665		
T3	0.1127	0.06379		

**Table:** (b) Pair-wise comparison of mean white spot lesions (ICDAS scores) in group A (NaF) using LSD-Bonferroni test

(I) Time	(J) Time	Mean difference (I-J)	Std. error	*p* value	95% confidence interval for difference	
					Lower bound	Upper bound
T0	T1	-.026^∗^	0.006	<0.001^∗^	-0.042	-0.01
	T2	-.068^∗^	0.009	<0.001^∗^	-0.092	-0.044
	T3	-.088^∗^	0.012	<0.001^∗^	-0.121	-0.055

T1	T2	-.042^∗^	0.007	<0.001^∗^	-0.062	-0.023
	T3	-.062^∗^	0.011	<0.001^∗^	-0.093	-0.03

T2	T3	-0.019	0.008	0.093	-0.041	0.002

**(a):** 

	Mean (*n* = 45)	Std. deviation	ANOVA *F* value	*p* value
T0	0.014	0.024	7.923	0.003^∗^
T1	0.022	0.026		
T2	0.038	0.041		
T3	0.015	0.027		

**Table:** (b) Pair-wise comparison of mean white spot lesions (ICDAS scores) in group B (APF)—LSD-Bonferroni test

(I) Time	(J) Time	Mean difference (I-J)	Std. error	*p* value	95% confidence interval for difference	
					Lower bound	Upper bound
T0	T1	-.008^∗^	0.002	0.009^∗^	-0.015	-0.001
	T2	-.024^∗^	0.007	0.01^∗^	-0.044	-0.004
	T3	-0.001	0.006	0.912	-0.017	0.015

T1	T2	-0.016	0.007	0.178	-0.036	0.004
	T3	0.007	0.006	0.916	-0.009	0.024

T2	T3	.024^∗^	0.004	0.001^∗^	0.014	0.033

ICDAS (International Caries Detection and Assessment System) index; T0: 0; T1: 4 weeks; T2: 12 weeks; T3: 24 weeks. ^∗^*p* < 0.05 (significant); *p* > 0.05 (not significant).

**(a):** 

	Mean (*n* = 45)	Std. deviation	ANOVA *F* value	*p* value
T0	0.08	0.019	21.172	0.001
T1	0.22	0.185		
T2	0.339	0.194		
T3	0.477	0.566		

**Table:** (b) Pair-wise comparison of mean GI scores in group A (NaF)—LSD-Bonferroni test

(I) Time	(J) Time	Mean difference (I-J)	Std. error	*p* value	95% confidence interval for difference	
					Lower bound	Upper bound
T0	T1	-.212^∗^	0.028	0.001^∗^	-0.29	-0.134
	T2	-.331^∗^	0.03	0.001^∗^	-0.415	-0.247
	T3	-.469^∗^	0.085	0.001^∗^	-0.704	-0.234

T1	T2	-.119^∗^	0.034	0.006^∗^	-0.213	-0.026
	T3	-.257^∗^	0.082	0.018^∗^	-0.483	-0.031

T2	T3	-0.138	0.075	0.427	-0.344	0.068

^∗^ *p* < 0.05 (significant); *p* > 0.05 (not significant). GI: gingival index of Loe and Silness; T0: 0; T1: 4 weeks; T2: 12 weeks; T3: 24 weeks.

**(a):** 

	Mean (*n* = 45)	Std. deviation	ANOVA *F* value	*p* value
T0	0.003	0.01	32.976	0.001^∗^
T1	0.136	0.12		
T2	0.181	0.091		
T3	0.097	0.114		

**Table:** (b) Pair-wise comparison of mean GI scores in group B (APF)—LSD-Bonferroni test

(I) time	(J) time	Mean difference (I-J)	Std. error	*p* value	95% confidence interval for difference	
					Lower bound	Upper bound
T0	T1	-.133^∗^	0.018	0.001^∗^	-0.183	-0.083
	T2	-.133^∗^	0.018	0.001^∗^	-0.183	-0.083
	T3	-.133^∗^	0.018	0.001^∗^	-0.183	-0.083

T1	T2	-0.045	0.022	0.245	-0.105	0.014
	T3	0.039	0.025	0.781	-0.031	0.108

T2	T3	.084^∗^	0.013	0.001^∗^	0.048	0.12

^∗^ *p* < 0.05 (significant); *p* > 0.05 (not significant). GI: gingival index of Loe and Silness; T0: 0; T1: 4 weeks; T2: 12 weeks; T3: 24 weeks.

**Table 9:** Intergroup comparison of mean severity white spot lesions (ICDAS scores) at different time intervals using unpaired *t*-test.

	Groups	Mean (*n* = 45)	Std. deviation	Mean difference	Unpaired *t*-statistic	*p* value
T0	NaF	0.025	0.027	0.011	2.018	0.057
	APF	0.014	0.024			

T1	NaF	0.051	0.038	0.029	4.231	0.001^∗^
	APF	0.022	0.026			

T2	NaF	0.093	0.047	0.055	5.91	0.001^∗^
	APF	0.038	0.041			

T3	NaF	0.113	0.064	0.098	9.466	0.001^∗^
	APF	0.015	0.027			

^∗^ *p* < 0.05 (significant); *p* > 0.05 (not significant).

**Table 10:** Intergroup comparison of mean GI scores at different time intervals using unpaired *t*-test.

	Groups	Mean (*n* = 45)	Std. deviation	Mean difference	Unpaired *t*-statistic	*p* value
T0	NaF	0.08	0.019	0.06	1.713	0.09
	APF	0.03	0.01			

T1	NaF	0.22	0.185	0.084	2.563	0.012^∗^
	APF	0.136	0.12			

T2	NaF	0.339	0.194	0.158	4.937	0.001^∗^
	APF	0.181	0.091			

T3	NaF	0.477	0.566	0.38	4.418	0.001^∗^
	APF	0.097	0.114			

^∗^ *p* < 0.05 (significant); *p* > 0.05 (not significant).

## Data Availability

The data supporting the findings of this research can be obtained directly from the authors of the study.

## Abbreviations

APF: Acidulated phosphate fluoride
CaF2: Calcium fluoride
CPP-ACP: Casein phosphopeptide amorphous
FHA: Fluorhydroxyapatite
GI: Gingival index
HA: Hydroxyapatite
ICDAS: International Caries Detection and Assessment System
NaF: Sodium fluoride
QLF: Quantitative laser fluorescence
WSL: White spot lesions.
